# CD79B in myelodysplastic syndromes and acute myeloid leukemia: an integrative computational and *in vitro* study

**DOI:** 10.3389/fmed.2025.1650035

**Published:** 2026-01-16

**Authors:** Xiangjing Kong, Yongfu Wei, Shengjuan Zhang, Xiaoya Lu, Rui Luo, Bo Liang, Yongsheng Chen

**Affiliations:** 1 Department of Hematology, The Second People’s Hospital of Nanning City, Nanning, Guangxi Zhuang Autonomous Region, China; 2 Department of Blood Transfusion, The Second People’s Hospital of Nanning City, Nanning, Guangxi Zhuang Autonomous Region, China; 3 Department of Scientific Research Project, Wuhan Kindstar Medical Laboratory Co., Ltd., Wuhan, China; 4 Department of Hematology, The Second Affiliated Hospital of Guangxi Medical University, Nanning, China

**Keywords:** acute myeloid leukemia, CD79b, cell cycle, immune-related genes, myelodysplastic syndromes

## Abstract

**Objectives:**

CD79B is a key component of the B-cell receptor complex, but its relevance in myelodysplastic syndromes (MDS) and acute myeloid leukemia (AML) remains unclear.

**Methods:**

We screened immune-related genes in public MDS microarray datasets, prioritized CD79B, and validated its expression in an independent MDS cohort, an AML cohort, and peripheral blood samples from patients with MDS or AML transformed from MDS. Functional effects of CD79B overexpression were examined in HL-60 cells, and gene set enrichment and immune-infiltration analyses were used to explore CD79B-associated pathways.

**Results:**

CD79B expression was consistently reduced in MDS and AML compared with normal controls in public datasets and clinical samples. In HL-60 cells, enforced CD79B expression modestly altered cell-cycle distribution and increased apoptosis. Transcriptomic analyses linked higher CD79B expression to immune response and T-cell activation pathways and to global patterns of immune-cell infiltration.

**Conclusion:**

These exploratory data suggest that CD79B downregulation is a recurrent feature of MDS and AML and that CD79B may influence leukemic cell behavior and immune microenvironmental signals. The findings generate hypotheses for future mechanistic studies and evaluation of CD79B as a potential biomarker in myeloid malignancies.

## 1 Introduction

Myelodysplastic syndromes (MDS) are heterogeneous clonal myeloid neoplasms arising from hematopoietic stem cells. They are characterized by ineffective hematopoiesis, which leads to refractory cytopenias involving the erythroid, granulocytic, and megakaryocytic lineages, bone marrow failure, and a substantial risk of progression to acute myeloid leukemia (AML) (1, 2). The incidence of MDS is very low in children and young adults (0.1/100,000 person-years in individuals <40 years) but increases sharply with age, reaching 30.2/100,000 in those aged 70–79 years and 59.8/100,000 in individuals ≥80 years. The median age at diagnosis is 70–75 years. Primary MDS accounts for approximately 80–90% of cases and typically arises in older adults without a clearly identifiable history of cytotoxic exposure (3, 4). Secondary MDS, which represents the remaining 10–20% of cases, usually develops after chemotherapy, radiotherapy, or chronic environmental insults and is frequently associated with complex cytogenetic abnormalities and a more aggressive clinical course. In the present study, we use the term primary MDS to denote cases without documented prior cytotoxic or environmental exposure, whereas secondary MDS refers to therapy-related or exposure-related disease, which typically exhibits more complex karyotypes and poorer clinical outcomes. AML is another malignant clonal hematologic disorder characterized by marked molecular heterogeneity (5, 6). These and related clonal diseases are driven by dysregulated differentiation and uncontrolled proliferation of hematopoietic progenitor cells, which often give rise to diverse cytogenetic abnormalities (7).

Both MDS and AML are hematologic malignancies arising from clonal hematopoietic stem and progenitor cells and they share many recurrent cytogenetic and molecular abnormalities (8, 9). Like AML, MDS is a genetically heterogeneous clonal stem-cell disorder with substantial overlap in driver mutations and cytogenetic lesions. Clinically, MDS is dominated by ineffective hematopoiesis and limited proliferative capacity, whereas AML is characterized by uncontrolled blast expansion and aggressive dissemination. Thus, the two entities differ in the degree of blast accumulation, the severity of differentiation arrest, and their overall clinical aggressiveness. In many patients with lower-risk MDS, increased apoptosis and peripheral cytopenias predominate, whereas progression to AML is defined by expansion of blasts to ≥20% in the bone marrow or peripheral blood together with rapidly progressive disease (5–7, 10). To improve treatment outcomes across this disease spectrum, it is crucial to elucidate the biological mechanisms that initiate MDS and drive its progression to AML and to identify novel therapeutic targets.

The risk of AML is markedly increased in patients with MDS, suggesting that specific susceptibility factors within the clonal MDS compartment and its microenvironment promote leukemic transformation (11). Persistent activation of inflammatory pathways, dysregulated innate and adaptive immunity, and genetic susceptibility are thought to create a permissive milieu for disease progression (12, 13). Mechanistically, chronic inflammatory signaling, immune evasion through inhibitory checkpoints, functional exhaustion of T cells, and impaired antigen presentation by dendritic cells and other antigen-presenting cells all contribute to this dysregulated immune milieu. The bone marrow immune microenvironment—including altered T-cell function, myeloid-derived cells, and antigen-presenting cells—is therefore pivotal in both MDS and AML (14). Consequently, a deeper understanding of how immune dysregulation intersects with clonal evolution in MDS and AML, particularly in patients who progress to AML, is essential for the development of new therapeutic strategies.

Previous transcriptomic studies of MDS and AML, including meta-analyses of public datasets (15, 16), have already reported downregulation of B-cell lineage markers such as CD79B in myeloid samples, largely reflecting reduced B-cell representation in these disorders. Our study therefore does not claim novelty for the simple observation of CD79B downregulation; instead, we aim to integrate public datasets with *in vitro* experiments to explore the potential functional and immunological implications of CD79B in MDS and AML.

Accordingly, we designed this study to (i) systematically screen immune-related genes in MDS to identify candidates that are consistently dysregulated across independent cohorts, (ii) prioritize CD79B as a shared B-cell-associated gene with concordant downregulation in both MDS and AML, and (iii) integrate public transcriptomic data with clinical specimens and *in vitro* AML cell experiments to investigate the potential functional and immunological roles of CD79B across this disease spectrum.

## 2 Materials and methods

### 2.1 Data collection and preprocessing

Three independent gene expression profiles, GSE58831, GSE19429 and GSE30029, were obtained from the Gene Expression Omnibus (GEO) database. In this study, gene expression profiling was performed on bulk hematopoietic cell fractions (including bone marrow CD34-based samples in the public datasets and PBMNCs in our small clinical cohort) rather than on immunophenotypically purified leukemic blasts, because flow-cytometric measurements of CD79B on blast populations were not available in the public datasets or in our experimental cohort. Specifically, three GEO datasets were used: GSE58831 (GPL570 platform: 159 MDS and 17 matched normal samples), GSE19429 (GPL570 platform: 183 MDS and 17 matched normal samples) and GSE30029 (GPL6947 platform: 90 AML and 31 matched normal samples). In these public microarray datasets, gene expression profiles were generated from bone marrow CD34-based hematopoietic cell fractions rather than from immunophenotypically purified leukemic blasts; therefore, decreases in CD79B signal may partly reflect reduced representation of B-cell lineage components rather than blast-intrinsic downregulation. The inclusion criteria for GEO datasets were: (a) availability of normal control samples, (b) patients clearly diagnosed with MDS or AML and (c) complete gene-expression data for the included samples. Exclusion criteria were: (a) metastatic tissue samples and (b) samples lacking expression data. Risk-stratification information (for example, IPSS/IPSS-R categories) was not uniformly available across the included datasets; therefore, analyses were not stratified by clinical risk groups. Similarly, detailed cytogenetic profiles and recurrent gene mutations with prognostic significance were incompletely annotated, precluding systematic correlation of CD79B expression with specific cytogenetic or molecular subgroups. Information on prior MDS history was also limited, and we could not reliably distinguish secondary AML evolving from MDS from *de novo* AML cases within the public AML datasets. Raw microarray data were analyzed using R software (version 4.2.1) to evaluate gene-expression levels and prognosis (17). In addition, 1,754 immune-related genes (IRGs) were retrieved from the published literature (18). For each GEO series, raw CEL files or series matrix files were downloaded and processed in R. When raw data were available, probes were background-corrected, quantile-normalized and summarized to gene-level expression values using standard Bioconductor pipelines (affy/limma). When only pre-processed matrices were available, we used the normalized expression values provided by GEO. All analyses were performed separately within each dataset, rather than by merging across different platforms, thereby avoiding the need for explicit cross-platform batch-effect correction. Detailed clinical and disease characteristics of the public cohorts, including information on *de novo* versus secondary AML, are described in the original publications for GSE30029 (19, 20), GSE58831 (21) and GSE19429 (22, 23). In our analyses, we did not perform separate stratified analyses for *de novo* versus secondary AML because this information was not uniformly available at the individual-sample level in the downloaded expression matrices. Thus, GSE58831 and GSE19429 primarily represent bone marrow CD34-enriched hematopoietic progenitor cells, whereas GSE30029 and our small clinical cohort reflect unfractionated PBMNC samples, which should be considered when interpreting differences in CD79B expression.

### 2.2 IRG expression and functional/pathway enrichment analysis in MDS

IRG expression levels in MDS were evaluated using GSE58831. Differentially expressed genes were identified using fold change and multiple-testing-adjusted *p*-values (FC >1.50 or <0.67, adjusted *p* < 0.05). We chose to apply absolute fold-change thresholds rather than log2-transformed thresholds because FC values are directly interpretable while being mathematically equivalent to |log2FC| >0.58; combined with adjusted *p* < 0.05, this conservative criterion helps to limit spurious differential-expression calls in these relatively small microarray cohorts. Functional clustering analysis of gene and protein sets was conducted using Metascape (24).

### 2.3 Validation of IRG expression in MDS

To validate key IRG expression, GSE58832 was used for expression analysis. Following data standardization, we assessed batch effects to verify data quality. Only datasets that demonstrated no evidence of batch-related clustering during quality-control assessment were included in subsequent analyses. In these analyses, MDS samples were coded in red and healthy control samples in blue (25).

### 2.4 Validation of CD79B expression in MDS and MDS-to-AML clinical samples

CD79B was selected for validation. A total of nine subjects were enrolled, comprising six patients (three with MDS and three with AML transformed from antecedent MDS, hereafter referred to as MDS-to-AML) and three healthy volunteers. Clinical samples were obtained from the Departments of Hematology at the Second Affiliated Hospital of Guangxi Medical University and the Second People’s Hospital of Nanning City, Guangxi Province. The study was approved by the Ethics Committee of the Second Affiliated Hospital of Guangxi Medical University (Approval Number: 2020-KY (0159)). Written informed consent was obtained from all participants before enrollment. Peripheral blood mononuclear cells (PBMNCs) were collected from each participant before initial treatment, and total RNA was extracted using TRIzol reagent (Vazyme, Cat: R401-01). Reverse transcription quantitative PCR (RT-qPCR) was performed using HiScript III RT SuperMix and ChamQ Universal SYBR qPCR Master Mix (both Vazyme). Reactions were run on a LightCycler^®^ 480 II Real-Time PCR System (Roche), with GAPDH as the internal reference. CD79B relative expression levels were quantified using the 2^−ΔΔCT^ method. Primer sequences are shown in Table 1. Detailed clinical characteristics of the six patients with MDS or MDS-to-AML and the three healthy donors—including diagnosis, blast percentage, cytogenetic risk category and treatment status at the time of sampling—are summarized in Table 2 (“Clinical characteristics of patients”). Fold-change values were compared between groups using the Wilcoxon rank-sum test (26).

**Table 1:** Primer sequence.

Name	Primer	Sequence
GAPDH	Forward	TCAAGAAGGTGGTGAAGCAGG
	Reverse	TCAAAGGTGGAGGAGTGGGT
CD79B	Forward	CTTCATCATCGTGCCTATCTT
	Reverse	CACTTCACTTCCCCTGTCC

**Table 2:** Clinical characteristics of patients.

Sample ID	Sex	Age (years)	Diagnosis subtype and risk	Cytogenetic profile/FISH	Bone marrow blasts (%)	Mutational background	Treatment status at sampling
1	Male	67	MDS with ring sideroblasts (ring sideroblasts 18%; IPSS-R score 4, intermediate risk)	FISH: trisomy 8; karyotype 47, XY, +8 [17]/46, XY [3]	0	No gene mutations detected	Sample collected after three cycles of lenalidomide, in partial remission (PR)
2	Female	75	MDS with excess blasts-2 (IPSS score 2, intermediate-2; WPSS score 3, high risk; IPSS-R score 5.5, high risk)	Normal karyotype	4.5	Not performed	Sample collected after four cycles of azacitidine; bone marrow blasts 6.5% at assessment
3	Male	72	MDS, refractory anemia	Not performed	3	Not performed	Supportive transfusion only
4	Female	69	AML transformed from MDS with IDH1 and TET2 mutations, poor-risk group	46, XX, +1,der(1;22)(q10;q10)[5]/46,idem,−21,+mar[5]/46, XX[10]	47.5	IDH1 and TET2 mutations	Sample collected after first cycle of IA chemotherapy; complete remission (CR)
5	Male	72	AML transformed from MDS with DNMT3A, PPMID and TP53 mutations, complex karyotype, poor-risk group	add(3q), add(5q) (5q−), add(7q) (7q−), +8	25	DNMT3A, PPMID and TP53 mutations	Sample collected after three cycles of VA chemotherapy; no remission (NR)
6	Male	53	AML transformed from MDS, poor-risk group	43–44, X,-Y,−7,psu dic(16;18)(p13;p11),−19,add(21)(p11),+r,inc[20]	37	Not performed	Sample collected after second cycle of VA chemotherapy; no further evaluation, treatment discontinued

a) Numbers in square brackets in the karyotype column (for example, [17] or [3]) indicate the number of metaphases showing the indicated karyotype out of 20 metaphases examined.

b) Cytogenetic symbols follow the International System for Human Cytogenomic Nomenclature (ISCN): “+” indicates gain of a whole chromosome or segment, “−” indicates loss, and add(5q)/add(7q) denote additional material of unknown origin on the long arm of chromosomes 5 and 7, consistent with 5q− and 7q− abnormalities.

c) “Not performed” in the mutational background column indicates that targeted myeloid gene mutation testing was not carried out for that patient at the time of sampling.

d) PR, partial remission; CR, complete remission; NR, no remission. IA and VA indicate standard induction chemotherapy regimens used at our institution; detailed drug combinations are recorded in the clinical treatment protocols.

### 2.5 Validation of CD79B expression in AML

CD79B expression was further validated using GSE30029. Following data standardization, we assessed batch effects to verify data quality. Only datasets that demonstrated no evidence of batch-related clustering during quality-control assessment were included in subsequent analyses. In these analyses, AML samples were coded in red and healthy control samples in blue (27).

### 2.6 Construction of AML cell models with CD79B overexpression

HL-60 cells were obtained from the cell bank at Nanning Second People’s Hospital, Guangxi Province. Cells were cultured in RPMI-1640 medium (HyClone, Cytiva) supplemented with 10% fetal bovine serum (Gibco, Thermo Fisher Scientific), 100 U/mL penicillin and 100 μg/mL streptomycin, and maintained at 37 °C in a humidified incubator with 5% CO₂. For CD79B overexpression experiments, HL-60 cells in logarithmic growth phase were seeded so that cell density was approximately 70% at the time of transfection and then divided into an empty-vector control group (HL-60+NC) and a CD79B-overexpression group (HL-60+CD79B-OE). The CD79B overexpression vector and the corresponding empty vector were transfected into HL-60 cells according to the manufacturer’s instructions, and CD79B overexpression was subsequently confirmed by RT-qPCR using the same reagents and cycling conditions as described for patient samples in Section 2.4 (28). All cell-culture and transfection experiments were performed with cells in logarithmic growth phase and were repeated in three independent biological replicates. After 48 h of transfection, when cell density had reached approximately 90%, cells were harvested for subsequent cell-cycle and apoptosis assays. HL-60 cells were handled according to the standard operating procedures of the Nanning Second People’s Hospital cell bank, including routine authentication and mycoplasma testing, to ensure experimental reproducibility.

### 2.7 Cell-cycle experiment

Cells were trypsinized, neutralized with culture medium and pelleted by centrifugation (1,000 rpm, 5 min). The pellet was resuspended in 5 mL of pre-chilled 70% ethanol, gently mixed and fixed overnight at 4 °C. After fixation, cells were collected by centrifugation (1,000 rpm, 5 min), washed twice with 5 mL PBS and resuspended in 500 μL permeabilization/staining solution containing 100 μg/mL RNase A and 0.2% Triton X-100. The suspension was incubated at 37 °C for 30 min, after which propidium iodide (50 μg/mL) was added and incubation continued for 10 min. Cells were then filtered through a 300-mesh sieve, centrifuged (1,000 rpm, 5 min) and the supernatant discarded. Finally, cells were washed twice with 5 mL PBS to remove residual propidium iodide, resuspended in 200 μL PBS and analyzed by flow cytometry (29). A commercial cell-cycle detection kit (Solarbio, Beijing, China) was used according to the manufacturer’s instructions.

### 2.8 Cell apoptosis experiment

Approximately 1 × 10^6^ cells were harvested and washed once with chilled PBS. The pellet was resuspended in 1 mL of 1× binding buffer to obtain a concentration of 1 × 10^6^ cells/mL. For each sample, 100 μL of cell suspension (1 × 10^5^ cells) was transferred to a tube, followed by the addition of 5 μL Annexin V-FITC. The mixture was gently vortexed and incubated at room temperature in the dark for 10 min. Subsequently, 5 μL propidium iodide was added and incubation continued for another 5 min in the dark. Finally, 400 μL of 1× binding buffer was added to each tube to a final volume of 500 μL. Samples were gently mixed and analyzed by flow cytometry within 1 h (30). An Annexin V-FITC/PI apoptosis detection kit (Solarbio, Beijing, China) was used according to the manufacturer’s protocol.

### 2.9 Transcriptional pathway analysis of CD79B in MDS and AML

To identify CD79B-associated transcriptional pathways in MDS and AML, genes showing a significant correlation with CD79B (*p* < 0.05) were screened from the GSE58831 and GSE30029 datasets. In GSE58831, 432 genes were positively and 41 negatively associated with CD79B, whereas in GSE30029, 362 genes were positively and 305 negatively associated. Gene Set Enrichment Analysis (GSEA) was performed using the R package clusterProfiler (31). Genes were ranked according to their correlation with CD79B, and GSEA was conducted using Gene Ontology biological-process and KEGG pathway gene sets with 10,000 permutations. For each gene set, we obtained the normalized enrichment score (NES) and Benjamini–Hochberg-adjusted *p*-value. Immune-related pathways of interest were defined as gene sets with nominal *p* < 0.05, and the full ranked gene lists together with the complete GSEA output are provided in Supplementary Table 1.

### 2.10 Correlation of the immune microenvironment with CD79B in MDS and AML

For each dataset, a gene-expression distance matrix between samples was constructed using Euclidean distance based on CD79B expression, and the same distance metric was applied to construct distance matrices for the inferred infiltration levels of each immune-cell type in the GSE58831 (MDS) and GSE30029 (AML) datasets. Pearson correlation coefficients were then used to calculate the correlation between the two distance matrices and to obtain the Mantel statistic (Mantel’s *r*) and the corresponding *p*-value (Mantel’s *p*). These Mantel correlations were used to summarize the association between CD79B expression patterns and overall immune-cell infiltration profiles in each dataset.

### 2.11 Statistical analysis

Quantitative data are presented as mean ± standard deviation. All statistical analyses were conducted using R software and GraphPad Prism. Comparisons between two groups were performed using the Wilcoxon rank-sum test (wilcox.test in R) or Student’s *t*-test, as appropriate. A two-sided *p*-value <0.05 was considered statistically significant.

## 3 Results

### 3.1 IRG expression and functional/pathway enrichment analysis in MDS

**Figure 1:**
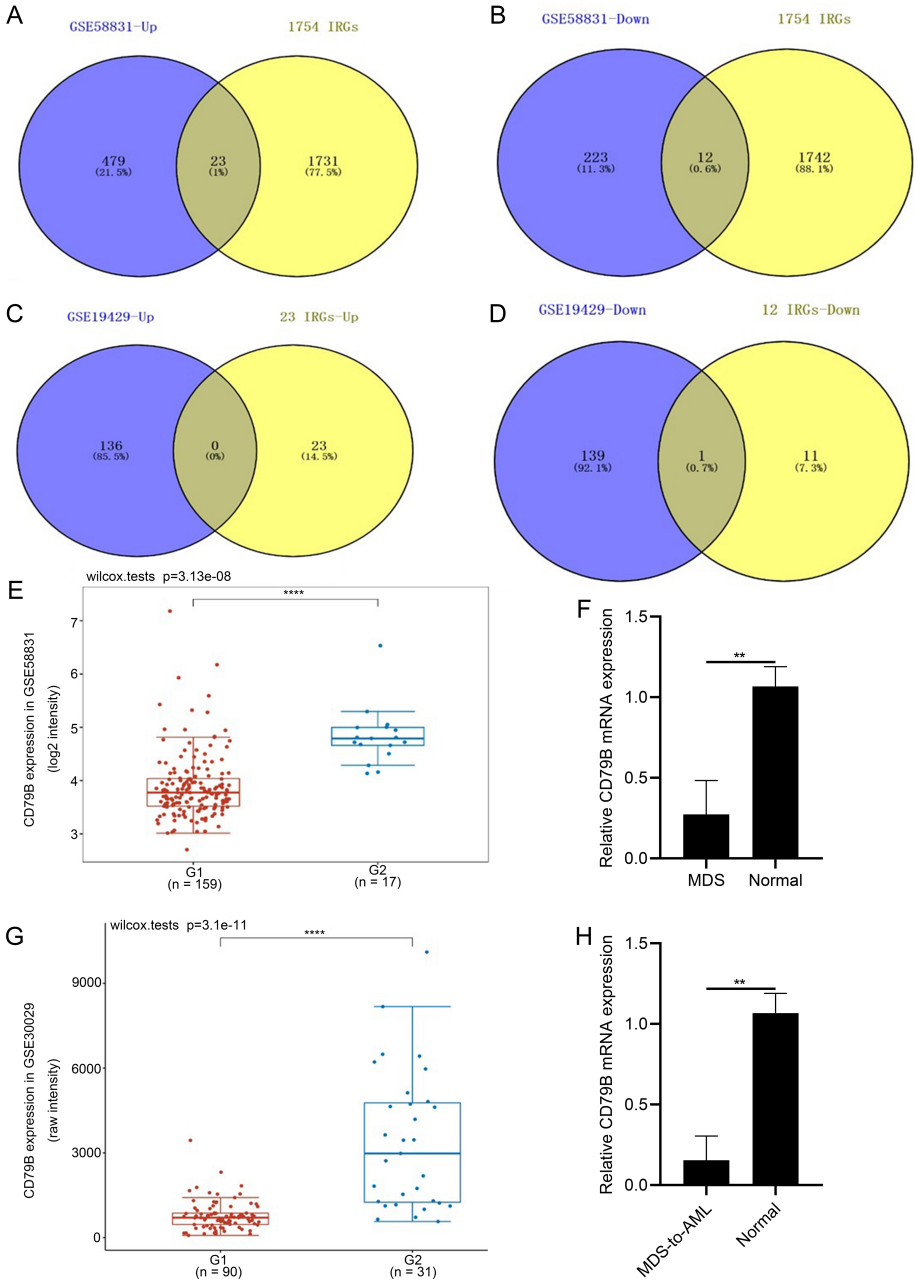
Validation of IRGs (CD79B) in datasets and clinical samples. A total of 35 IRGs were identified in the GSE58831 dataset, of which 23 were upregulated **(A)** and 12 were downregulated **(B)**. Based on the GSE19429 dataset, CD79B expression was significantly lower in MDS samples than in normal controls **(C,D)**. In the GSE58831 dataset, CD79B expression was likewise significantly downregulated in MDS samples compared with the normal group **(E)**. RT-qPCR analysis of clinical samples showed that CD79B mRNA levels were significantly reduced in MDS patients compared with healthy controls **(F)**. In the GSE30029 dataset, CD79B expression was significantly downregulated in AML samples relative to normal controls **(G)**. RT-qPCR results further demonstrated that CD79B expression was significantly decreased in patients with MDS-to-AML compared with the normal group **(H)**. In panel **E**, the *y*-axis shows normalized CD79B expression values from the GSE58831 dataset, and the *x*-axis shows the indicated groups [G1: MDS samples (*n* = 159), G2: normal samples (*n* = 17)]. In panel **F**, the *y*-axis shows relative CD79B mRNA expression measured by RT-qPCR (2^−ΔΔCT^), and the *x*-axis compares MDS patients (*n* = 3) with healthy controls (*n* = 3). In panel **G**, the *y*-axis shows normalized CD79B expression values from the GSE30029 dataset, and the *x*-axis shows the indicated groups [G1: AML samples (*n* = 90), G2: normal samples (*n* = 31)]. In panel **H**, the *y*-axis shows relative CD79B mRNA expression measured by RT-qPCR (2^−ΔΔCT^), and the *x*-axis compares MDS-to-AML patients (*n* = 3) with healthy controls (*n* = 3). Statistical methods: Wilcox test (panels **E,G**) and Student’s *t*-test (panels **F,H**). ^**^p < 0.01 and ^****^p < 0.0001.

A total of 35 immune-related genes were identified as differentially expressed when comparing MDS samples with normal controls in the GSE58831 dataset, with 23 genes upregulated in MDS and 12 downregulated (Figures 1A,B). These 35 IRGs were mainly enriched in GO and KEGG pathways related to cytokine-mediated signaling, regulation of immune responses, Th17 differentiation, PI3K-Akt signaling, MAPK signaling and T-cell receptor signaling (Supplementary Figure 1).

### 3.2 IRG expression validation in MDS patients and clinical samples

In the independent GSE19429 dataset, Venn diagrams confirmed that CD79B was retained as a shared downregulated immune-related gene (Figures 1C,D). Consistently, analysis of the independent GSE58831 dataset also demonstrated reduced CD79B expression in MDS compared with normal controls (Figure 1E). Subsequent RT-qPCR analysis of clinical specimens confirmed a marked decrease in CD79B expression in MDS cases relative to healthy donors (Figure 1F). Taken together, these findings indicate that CD79B expression is consistently lower in MDS than in normal controls across both public microarray datasets and our RT-qPCR validation cohort.

### 3.3 CD79B expression validation in MDS-to-AML using clinical samples

Analysis of the GSE30029 dataset showed significant downregulation of CD79B expression in AML samples compared with normal controls (Figure 1G). RT-qPCR results further demonstrated a substantial reduction in CD79B expression in patients with MDS-to-AML relative to the normal group (Figure 1H). In the figures, red denotes *de novo* AML samples or MDS-to-AML patients, whereas blue represents healthy control samples. Among the immune-related genes identified in the MDS datasets, CD79B was notable as a B-cell-associated receptor component that was consistently downregulated in both the discovery cohort GSE58831 and the independent validation cohort GSE19429, and it also showed reduced expression in the AML cohort GSE30029. On this basis, we selected CD79B for further validation and functional analyses.

### 3.4 Cell cycle analysis in HL-60 cells

**Figure 2:**
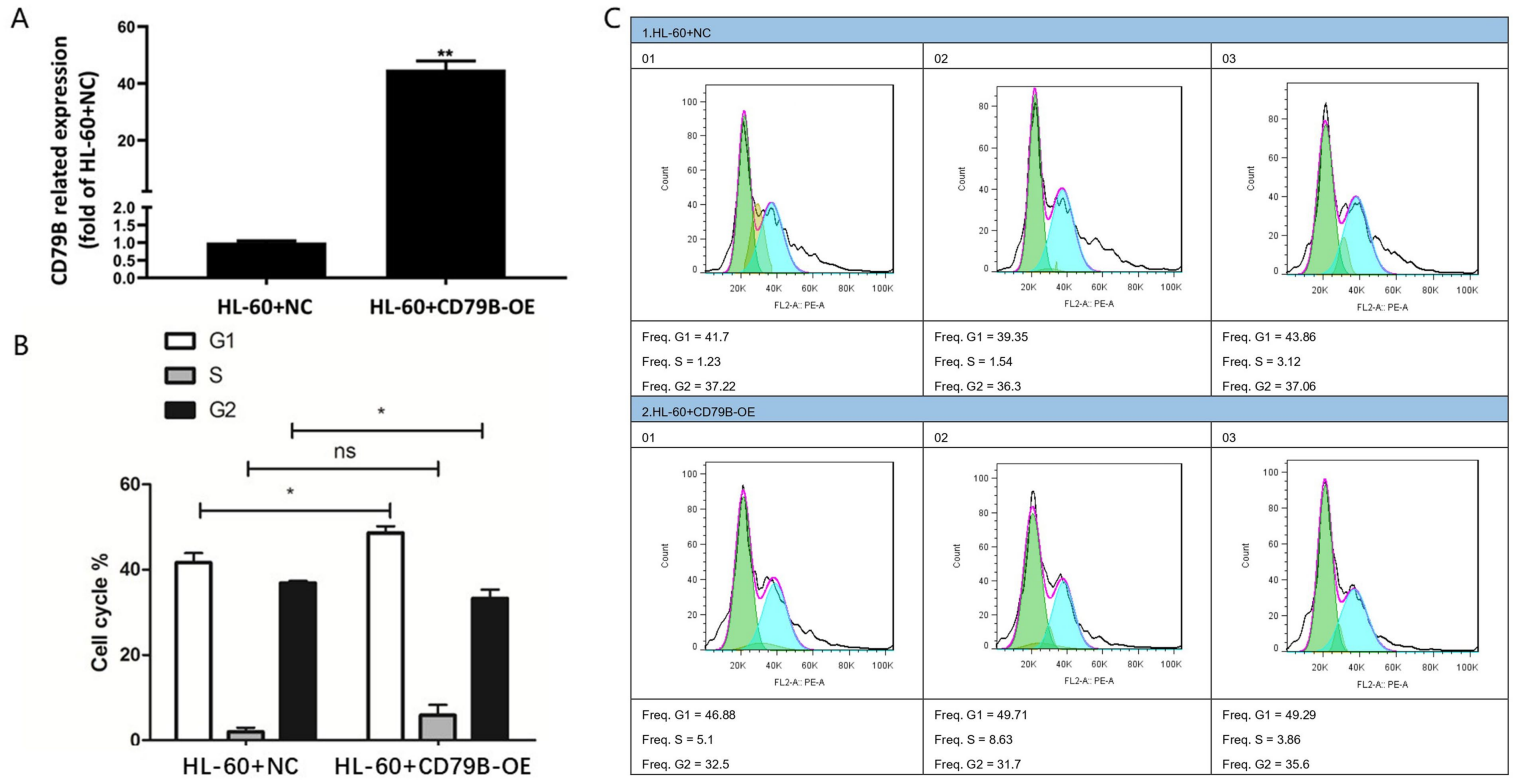
Construction of the CD79B-overexpressing HL-60 cell model and cell-cycle analysis. The RT-qPCR results showed that, compared with the control group, HL-60+CD79B-OE cells exhibited markedly increased CD79B expression **(A)**. Cell-cycle analysis demonstrated that, relative to HL-60+NC, HL-60+CD79B-OE cells showed a significant increase in the proportion of cells in G1 phase, an increase in S phase, and a significant decrease in G2 phase **(B,C)**. Bar graphs summarize the mean ± standard deviation from at least three independent experiments per group, and *p*-values for comparisons between HL-60+NC and HL-60+CD79B-OE are indicated in the figure. Comparisons between the two groups were performed using Student’s *t*-test. ^*^p < 0.05, ^**^p < 0.01, ^ns^*p* > 0.05.

RT-qPCR confirmed robust CD79B overexpression in HL-60+CD79B-OE cells compared with HL-60+NC controls (Figure 2A). These cells exhibited an increased proportion of cells in G1 and S phases and a pronounced reduction in G2 phase (Figures 2B,C). Collectively, these data indicate that CD79B overexpression is associated with a shift of HL-60 cells toward G1/S and away from G2, consistent with a modest slowing of cell-cycle progression.

### 3.5 Apoptosis analysis in HL-60 cells

**Figure 3:**
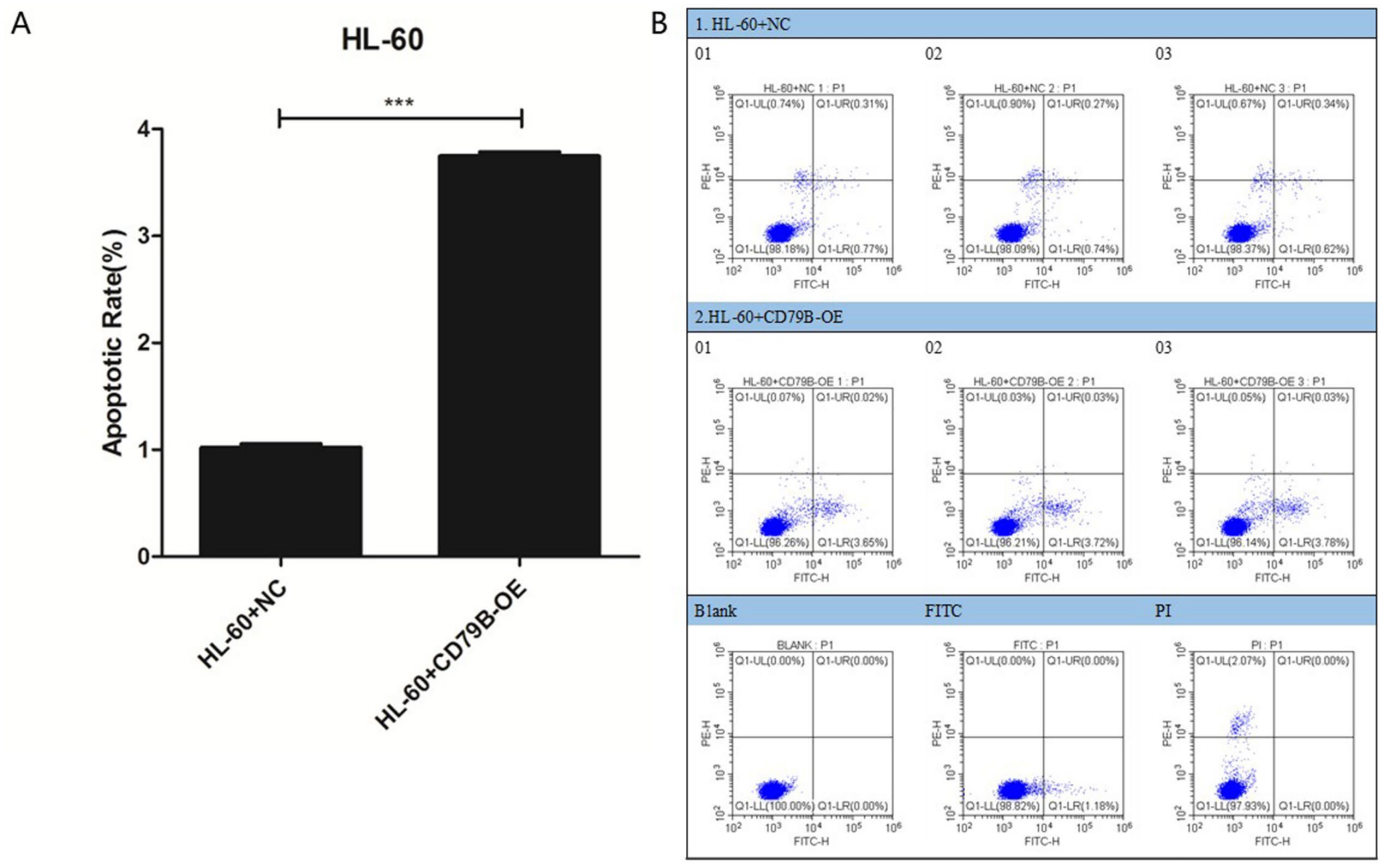
Apoptosis analysis of HL-60 cells. Flow-cytometric analysis showed that, compared with HL-60+NC cells, HL-60+CD79B-OE cells had a significantly higher apoptotic rate **(A,B)**. Bar graphs summarize the mean ± standard deviation from at least three independent experiments per group, and *p*-values for comparisons between HL-60+NC and HL-60+CD79B-OE are indicated in the figure. Comparisons between the two groups were performed using Student’s *t*-test. ^***^p < 0.05.

Compared with the HL-60+NC group, HL-60+CD79B-OE cells showed a significantly higher apoptosis rate (Figures 3A,B). These findings indicate that CD79B overexpression in HL-60 cells is associated with enhanced apoptotic activity.

### 3.6 Transcriptional pathway analysis of CD79B in MDS and AML

**Figure 4:**
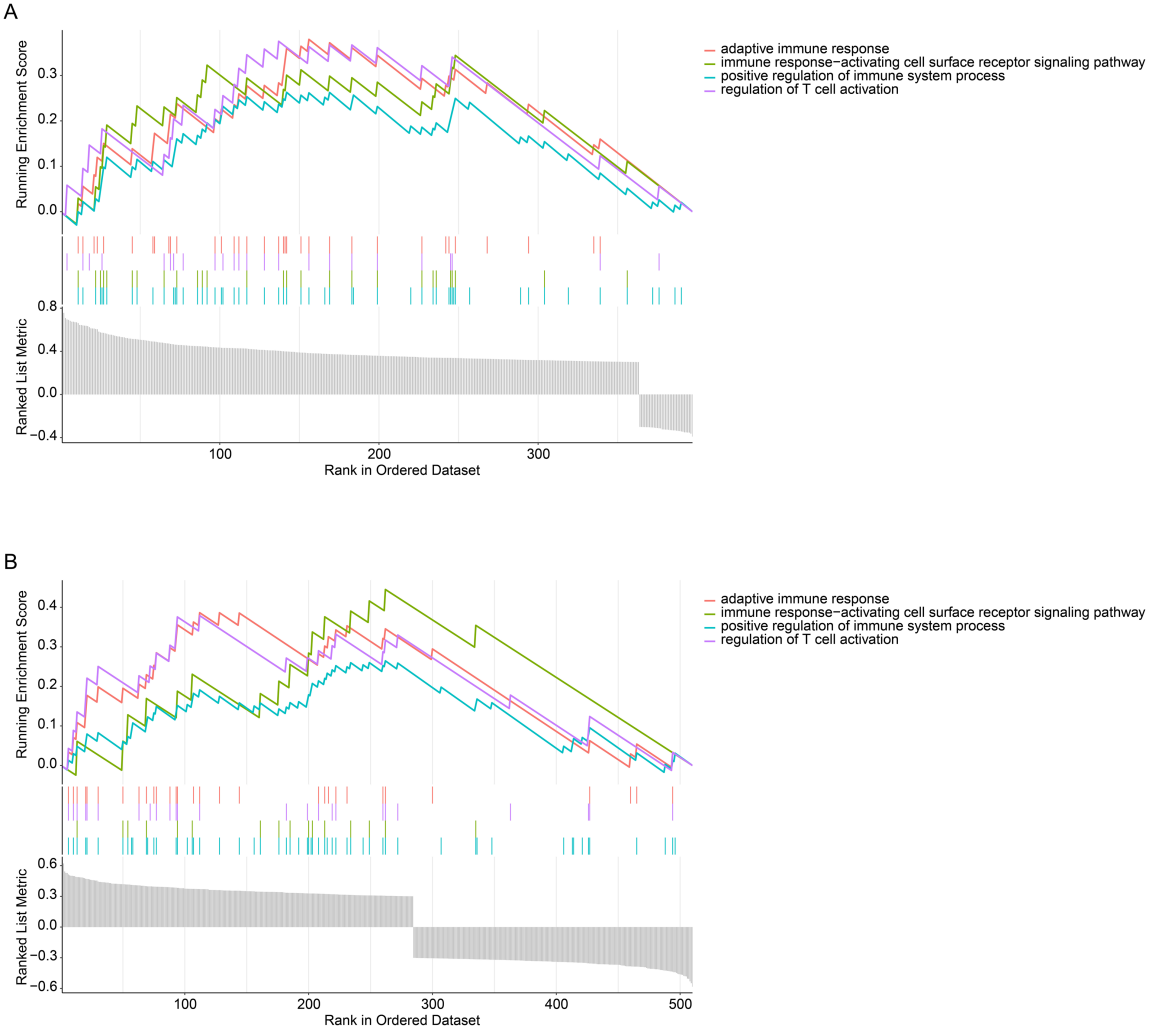
Pathway analysis of CD79B at the transcriptional level in the GSE58831 dataset **(A)** and GSE30029 dataset **(B)**. GSEA results indicate that higher CD79B expression is associated with activation of immune-related pathways, including positive regulation of immune system processes, immune response-activating cell-surface receptor signaling, regulation of T-cell activation, and adaptive immune responses. In both panels, the bottom gray bar plot displays the ranked CD79B-correlated gene list (*x*-axis), the upper curves show the running enrichment score for each immune-related gene set (*y*-axis), and the middle tick marks indicate the positions of pathway genes along the ranked list.

To identify CD79B-associated transcriptional pathways in MDS and AML, genes exhibiting a significant correlation with CD79B (*p* < 0.05) were screened from the GSE58831 (Figure 4A) and GSE30029 (Figure 4B) datasets. In GSE58831, 432 genes were positively and 41 negatively associated with CD79B, whereas in GSE30029, 362 genes were positively and 305 negatively associated. GSEA results indicated that higher CD79B expression was associated with enrichment of pathways involved in positive regulation of immune system processes, immune response-activating cell-surface receptor signaling, T-cell activation and adaptive immune responses. For each enriched pathway, the normalized enrichment score (NES) and false-discovery-rate-adjusted *p*-value are provided in Supplementary Table 1.

### 3.7 Correlation of CD79B with immune cell infiltration

**Figure 5:**
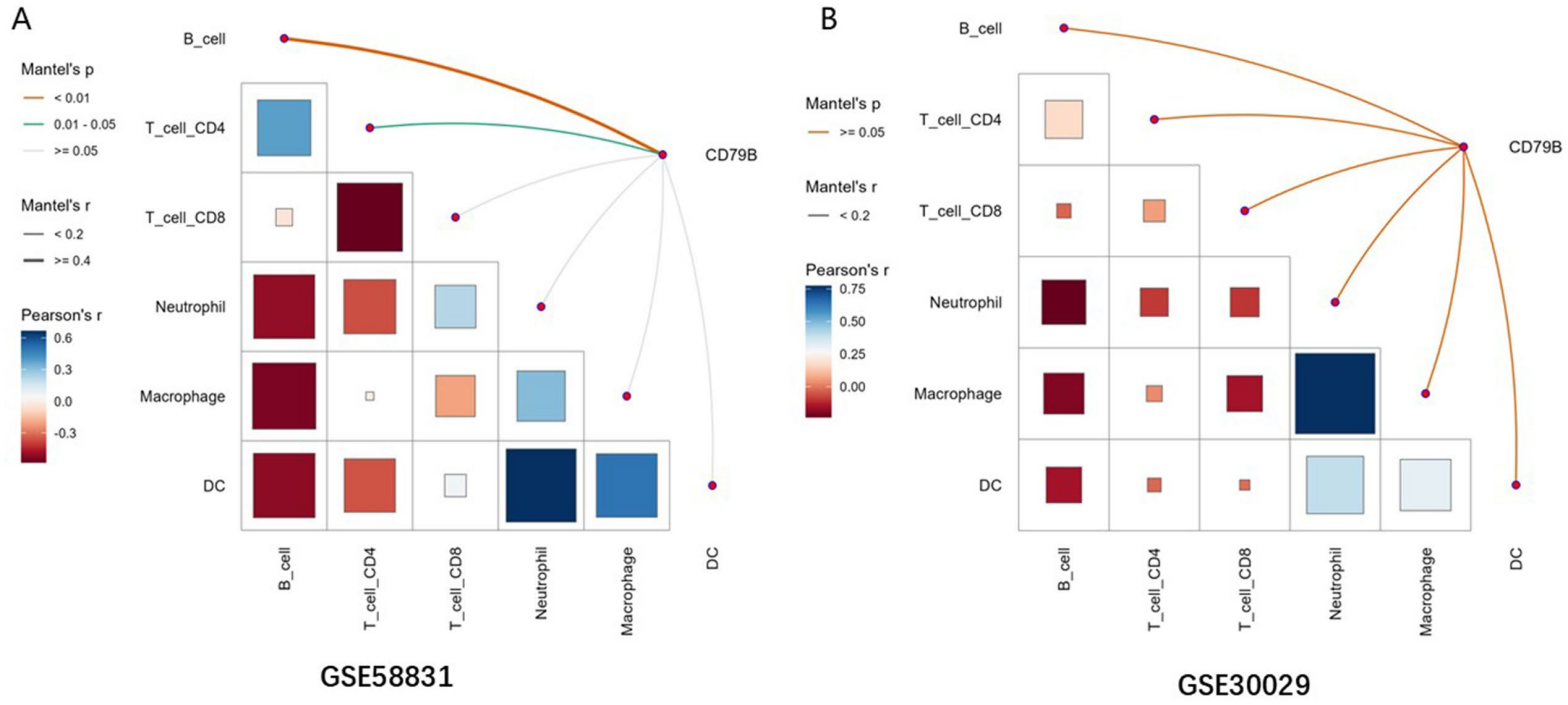
Correlation analysis between CD79B and immune-cell infiltration in the GSE58831 dataset **(A)** and GSE30029 dataset **(B)**. In both panels, the lower-left matrix shows pairwise Pearson correlation coefficients between immune-cell subsets (color scale and square size), and the axes list the evaluated immune cells (B cells, CD4^+^ T cells, CD8^+^ T cells, neutrophils, macrophages, and dendritic cells). The edges connecting CD79B to each immune cell encode Mantel’s *r* (line thickness) and Mantel’s *p*-value (edge color), summarizing the association between CD79B expression patterns and overall immune-cell infiltration profiles.

The TIMER method was applied to evaluate the relationship between CD79B expression and immune-cell infiltration in the GSE58831 (MDS) and GSE30029 (AML) datasets. In the GSE58831 dataset, CD79B expression showed significant Mantel correlations with the overall patterns of B-cell and CD4^+^ T-cell infiltration (Mantel’s *p* < 0.05), indicating that variation in CD79B tends to accompany broader changes in these immune-cell compartments rather than implying a simple positive or negative linear association with any single subset (Figure 5A). In contrast, in the GSE30029 dataset no significant Mantel correlations between CD79B and immune-cell infiltration profiles were observed (Mantel’s *p* ≥ 0.05); however, Pearson correlation analyses between immune-cell subsets themselves revealed a strong positive correlation between macrophages and neutrophils (Figure 5B).

## 4 Discussion

Previous studies have shown that patients with MDS have a markedly increased risk of developing AML and that immune dysregulation within the bone marrow microenvironment contributes to leukemic progression (32–34). Nevertheless, the shared molecular vulnerabilities that connect MDS and AML remain incompletely defined. Our study demonstrated that CD79B is significantly dysregulated in both MDS and AML. Moreover, functional experiments in the HL-60 AML cell line showed that enforced CD79B expression was associated with modest alterations in cell-cycle distribution and apoptosis. Importantly, these functional data were generated exclusively in a single AML cell line and therefore cannot be assumed to reflect CD79B biology in primary MDS blasts or in other AML subtypes; their interpretation should remain exploratory. In B-cell non-Hodgkin lymphomas such as diffuse large B-cell lymphoma and mantle cell lymphoma, activating mutations or overexpression of CD79B sustain B-cell receptor signaling and promote malignant B-cell survival (35–39). These observations support the concept that CD79B can participate in oncogenic signaling in lymphoid malignancies, although whether analogous mechanisms operate in myeloid neoplasms remains uncertain. Taken together, our *in vitro* findings support a possible role for CD79B in modulating leukemic cell behavior, but because they rely on a single AML cell line they should be regarded as hypothesis-generating rather than definitive mechanistic evidence for MDS-to-AML progression. The specific mechanisms by which CD79B might influence myeloid leukemogenesis therefore require further clarification.

GSEA indicated that higher CD79B expression is associated with positive regulation of immune system processes, T-cell activation and immune response-activating cell-surface receptor signaling in MDS and AML. Tumor-infiltrating B lymphocytes and plasma cells can mediate anti-tumor immunity through secretion of tumor-specific antibodies, thereby facilitating antibody-dependent cytotoxicity and promoting phagocytosis of tumor cells by phagocytes (40). Additionally, B cells directly or indirectly participate in antigen presentation and exhibit anti-tumor functions such as producing tumor-associated antibodies, promoting phagocytosis and presenting antigens to CD4^+^ T cells (41). In our deconvolution-based analyses of bulk transcriptomic data, CD79B expression did not show a simple one-to-one positive association with estimated B-cell abundance, and some Mantel correlations between CD79B and immune-cell infiltration patterns were modest or non-significant. This counterintuitive pattern likely reflects limitations of deconvolution in heterogeneous bone marrow samples, where technical noise, variation in myeloid and stromal compartments and reduced representation of normal B cells can obscure straightforward correlations (14, 42, 43). Therefore, reduced CD79B expression may contribute to an impaired anti-tumor immune response in patients with MDS and AML, but this hypothesis requires validation in functional studies of defined immune-cell subsets. Further research is needed to elucidate the specific mechanisms involved.

This study has several limitations that warrant cautious interpretation. Because transcriptomic profiling was performed on bone-marrow CD34^+^ fractions and PBMNCs rather than purified blasts or defined B-cell subsets, reduced CD79B may reflect shifts in cellular composition and/or cell-intrinsic regulation, which cannot be separated here. Our clinical validation cohort was small and CD79B was quantified in bulk PBMNCs; therefore, these findings should be considered exploratory pending confirmation in larger cohorts with blast-resolved measurements. Public datasets lacked uniformly available IPSS/IPSS-R and related risk information, precluding risk-stratified analyses. Cytogenetic and recurrent mutation annotations were incomplete and not harmonized, limiting molecular subgroup analyses. Secondary AML evolving from antecedent MDS could not be reliably distinguished from *de novo* AML in the public cohorts. Mechanistic work was performed in limited models and without direct interrogation of replication-stress/checkpoint signaling; broader validation and pathway mapping are needed. Enrichment analyses based on the 35 immune-related DEGs in GSE58831 should be interpreted as exploratory given the small input gene set. Finally, the limited RT-qPCR sample size prevented formal comparisons between MDS and MDS-to-AML, and future studies should jointly evaluate CD79A/CD79B in larger, well-annotated cohorts across the MDS-AML continuum. Despite these limitations, the convergent evidence from multiple independent transcriptomic cohorts together with our RT-qPCR validation supports that reduced CD79B expression is a reproducible feature across the MDS-AML continuum. Clinically, if confirmed in larger, well-annotated prospective cohorts with blast-resolved measurements, CD79B could be explored as an adjunct biomarker for disease characterization and longitudinal monitoring, and as a rationale to further interrogate B-cell-related immune signaling dysregulation in myeloid neoplasms.

## 5 Conclusion

This study confirmed that CD79B expression is reduced in MDS and AML compared with normal controls by integrating public transcriptomic datasets with a small cohort of clinical specimens, and it provided preliminary evidence from an AML cell line that modulation of CD79B can influence cell-cycle distribution and apoptosis. By combining GSEA with immune-infiltration analyses, we highlighted immune-related pathways that may be linked to CD79B expression across the MDS-AML spectrum. However, given the limited sample size, reliance on bulk expression data and absence of *in vivo* mechanistic experiments, these findings should be viewed as hypothesis-generating and position CD79B as a candidate biomarker and potential immunobiological modulator rather than an established therapeutic target.

## Data Availability

The original contributions presented in the study are included in the article/Supplementary material, further inquiries can be directed to the corresponding authors.

## Supplementary material

SUPPLEMENTARY FIGURE 1 Functional enrichment of 35 immune-related genes in GO and KEGG pathways (GSE58831 dataset) The *x*-axis represents −log10(*p*) for each enriched GO or KEGG term, and the *y*-axis lists the corresponding biological processes and pathways.
