# Supplementary figures and images for: CD79B in myelodysplastic syndromes and acute myeloid leukemia: an integrative computational and in vitro study
Source: Front Med (Lausanne). 2026 Jan 16;12:1650035. doi: 10.3389/fmed.2025.1650035 (PMC12855093; doi:10.3389/fmed.2025.1650035)

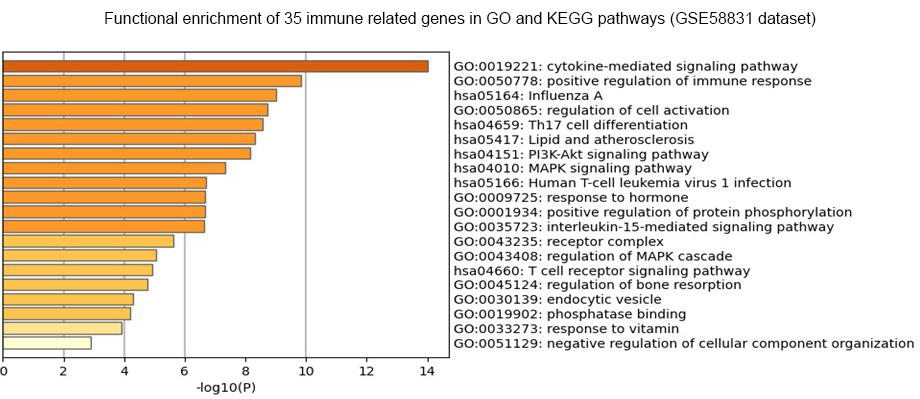

Supplement: SUPPLEMENTARY FIGURE 1 — Functional enrichment of 35 immune-related genes in GO and KEGG pathways (GSE58831 dataset) The x-axis represents −log10(p) for each enriched GO or KEGG term, and the y-axis lists the corresponding biological processes and pathways. [file Image_1.JPEG]
